# Supplementary material for: A retrospective analysis of postoperative hypokalemia in pituitary adenomas after transsphenoidal surgery
Source: PeerJ. 2017 May 23;5:e3337. doi: 10.7717/peerj.3337 (PMC5444367; doi:10.7717/peerj.3337)
Supplement: Supplemental Information 1 [file peerj-05-3337-s001.docx]

**Supporting Information**

S1 Table 1. Serum potassium in patients with hypokalemia at different time points

| Variables | Time points for monitoring serum potassium | | | |
| --- | --- | --- | --- | --- |
|  | T1 | T2 | T3 | T4 |
| Case#1 (control) | 3.16 | 4.16 | 4.33 | 4.56 |
| Case#2 (control) | 3.35 | 3.63 | 3.84 | 3.90 |
| Case#3 (control) | 3.44 | 3.59 | 3.76 | 3.91 |
| Case#4 (control) | 3.35 | 3.72 | 3.83 | 4.23 |
| Case#5 (control) | 3.34 | 3.79 | 4.12 | 4.44 |
| Case#6 (control) | 3.37 | 3.62 | 3.86 | 4.23 |
| Case#7 (control) | 3.32 | 3.66 | 4.02 | 4.44 |
| Case#8 (control) | 3.20 | 3.51 | 3.72 | 3.95 |
| Case#9 (ACTH) | 2.54 | 2.54 | 2.60 | 2.61 |
| Case#10 (ACTH) | 3.49 | 3.89 | 4.12 | 4.44 |
| Case#11 (ACTH) | 2.51 | 2.55 | 2.89 | 3.56 |
| Case#12 (ACTH) | 3.12 | 3.50 | 3.89 | 4.12 |
| Case#13 (ACTH) | 3.47 | 3.55 | 3.98 | 4.12 |
| Case#14 (ACTH) | 2.95 | 3.40 | 3.78 | 4.23 |
| Case#15 (ACTH) | 2.37 | 2.52 | 2.90 | 3.56 |
| Case#16 (ACTH) | 2.90 | 3.20 | 3.50 | 4.22 |

T1: day of surgery; T2: postoperative day 1; T3: postoperative day 2; T4: postoperative day 3.

S2 Table. Results of Mauchly’s test of sphericity

| Within subject effect | Mauchly’s W | Approx.  chi-square | df | *P* | Epsilon | | |
| --- | --- | --- | --- | --- | --- | --- | --- |
|  |  |  |  |  | Greenhouse–Geisser | Huynh–Feldt | Lower-bound |
| Time | 0.170 | 22.540 | 5 | 0.000 | 0.566 | 0.681 | 0.333 |

S3 Table. Tests of within subject effects for postoperative hypokalemia

| Source | Type III sum of squares | df | Mean square | *F* | *P* |
| --- | --- | --- | --- | --- | --- |
| Time |  |  |  |  |  |
| Sphericity assumed | 7.281 | 3 | 2.427 | 78.612 | <0.001 |
| Greenhouse–Geisser | 7.281 | 1.697 | 4.291 | 78.612 | <0.001 |
| Huynh–Feldt | 7.281 | 2.044 | 3.562 | 78.612 | <0.001 |
| Lower-bound | 7.281 | 1.000 | 7.281 | 78.612 | <0.001 |
| Time × group |  |  |  |  |  |
| Sphericity assumed | 0.108 | 3 | 0.036 | 1.166 | 0.334 |
| Greenhouse–Geisser | 0.108 | 1.697 | 0.064 | 1.166 | 0.321 |
| Huynh Feldt | 0.108 | 2.044 | 0.053 | 1.166 | 0.327 |
| Lower-bound | 0.108 | 1.000 | 0.108 | 1.166 | 0.298 |
| Error (Time) |  |  |  |  |  |
| Sphericity assumed | 1.297 | 42 | 0.031 |  |  |
| Greenhouse–Geisser | 1.297 | 23.758 | 0.055 |  |  |
| Huynh–Feldt | 1.297 | 28.622 | 0.045 |  |  |
| Lower-bound | 1.297 | 14.000 | 0.093 |  |  |

S4 Table. Tests of between-group effects

| Source | Type III sum of squares | df | Mean square | *F* | *P* |
| --- | --- | --- | --- | --- | --- |
| Intercept | 814.888 | 1 | 814.888 | 1428.481 | <0.001 |
| Group | 3.209 | 1 | 3.209 | 5.625 | 0.033 |
| Error | 7.986 | 14 | 0.570 |  |  |
